# Evaluation of a Rapid Diagnostic Test for Yaws Infection in a Community Surveillance Setting

**DOI:** 10.1371/journal.pntd.0003156

**Published:** 2014-09-11

**Authors:** Michael Marks, Adriana Goncalves, Ventis Vahi, Oliver Sokana, Elliot Puiahi, Zaixing Zhang, Tenneth Dalipanda, Christian Bottomley, David Mabey, Anthony W. Solomon

**Affiliations:** 1 Clinical Research Department, Faculty of Tropical and Infectious Diseases, London School of Hygiene & Tropical Medicine, London, United Kingdom; 2 Hospital for Tropical Diseases, University College London Hospitals NHS Trust, Mortimer Market, London, United Kingdom; 3 Solomon Islands Ministry of Health and Medical Services, Honiara, Solomon Islands; 4 World Health Organization, Western Pacific Region Office, Honiara, Solomon Islands; 5 Department of Infectious Diseases Epidemiology, London School of Hygiene & Tropical Medicine, London, United Kingdom; Kwame Nkrumah University of Science and Technology School of Medical Sciences, Ghana

## Abstract

Yaws is a non-venereal treponemal infection caused *by Treponema pallidum* ssp. *pertenue*. The WHO has launched a worldwide control programme, which aims to eradicate yaws by 2020. The development of a rapid diagnostic test (RDT) for serological diagnosis in the isolated communities affected by yaws is a key requirement for the successful implementation of the WHO strategy. We conducted a study to evaluate the utility of the DPP test in screening for yaws, utilizing samples collected as part of a community prevalence survey conducted in the Solomon Islands. 415 serum samples were tested using both traditional syphilis serology (TPPA and quantitative RPR) and the Chembio DPP Syphilis Screen and Confirm RDT. We calculated the sensitivity and specificity of the RDT as compared to gold standard serology. The sensitivity of the RDT against TPPA was 58.5% and the specificity was 97.6%. The sensitivity of the RDT against RPR was 41.7% and the specificity was 95.2%. The sensitivity of the DPP was strongly related to the RPR titre with a sensitivity of 92.0% for an RPR titre of >1/16. Wider access to DPP testing would improve our understanding of worldwide yaws case reporting and the test may play a key role in assessing patients presenting with yaws like lesions in a post-mass drug administration (MDA) setting.

## Author Summary

Yaws is a bacterial infection closely related to syphilis. The WHO has launched a worldwide campaign to eradicate yaws by 2020. If this goal is to be achieved, programme managers and clinical staff will need access to a rapid diagnostic test (RDT) for yaws that can be used in the remote communities where the disease is found. In this study, we present data evaluating one possible RDT for yaws as part of a community survey in the Solomon Islands. The test performed reasonably well—there were some false negatives but few false positives. The performance of the test was best in individuals with more active disease suggesting the test may be most appropriately used for confirming clinically diagnosed cases. These findings should prompt consideration of the use of this RDT as part of worldwide yaws control efforts.

## Introduction

Yaws is a non-venereal treponemal infection caused *by Treponema pallidum* ssp. *pertenue* (*T. pertenue*) [1] which is currently thought to be endemic in fourteen countries [2]. The emergence of azithromycin as an effective oral agent in the treatment of yaws [3] has prompted renewed calls for a coordinated worldwide programme to eradicate the disease by 2020 [4].

Despite this optimism there are significant barriers still to be overcome. The differential diagnosis of yaws can be broad [5] and serological testing is necessary to help establish a diagnosis. As with syphilis, latent infection occurs, and it is recognized that for every clinical case there may be 5–6 individuals with serological evidence of infection but no clinical manifestations [6], [7]. Failure to adequately treat latent cases was one of the reasons for the failure of previous attempts to eliminate yaws [8]. Detection and treatment of these latent cases will be extremely important if the WHO eradication programme is to achieve its target.

Traditional syphilis serology includes a treponemal specific test, such as the *Treponema pallidum* particle agglutination assay (TPPA) or the fluorescent treponemal antibody test, and a non-treponemal test, such as the Rapid Plasma Reagin (RPR) or Venereal Disease Research Laboratory (VDRL) assays. The former tests are highly specific but normally remain positive for life following infection. The latter tests are non-specific but the RPR titre more accurately reflects disease activity and falls following successful treatment. Low-titre false-positive RPRs may occur in a number of conditions including acute viral infections, malaria and connective tissue diseases. Testing therefore requires combined treponemal and non-treponemal assays to give a more accurate diagnostic result.

The development of a rapid diagnostic test (RDT) that can be used to improve access to serological diagnosis in the isolated communities affected by yaws has been highlighted as a major research question [9] to be addressed. RDTs allow wider access to diagnostic testing in remote communities where laboratory facilities are not available, with results available at the point of care to inform clinical decision making. As yaws is serologically indistinguishable from syphilis [10], the recent development of syphilis RDTs with high sensitivity and specificity [11] prompts evaluation of their use for the diagnosis of endemic treponemal diseases.

The required role(s) of an RDT may vary depending on the progress of the eradication programme in a given country. The test may have utility to confirm the diagnosis in patients presenting with skin lesions, to detect ongoing transmission of infection after mass drug administration has been conducted, or to conduct community surveillance in areas previously known to be endemic. The target product profiles of the RDTs required in each of these settings are likely to vary.

The Dual Path Platform (DPP) Syphilis Screen and Confirm (Chembio, Medford, NY, USA) provides both a “treponemal” result (analogous to a *Treponema Pallidum* Particle Agglutination (TPPA) assay (T1 line)) and a “non-treponemal” result (analogous to a qualitative Rapid Plasma Reagin (RPR) assay (T2 line)) [12]. We conducted a study to evaluate the utility of the DPP test in screening for yaws in the general population, utilizing samples collected as part of a community prevalence survey for yaws and trachoma conducted in the Solomon Islands.

## Methods

### Participant Recruitment

This study was embedded in a larger study investigating the epidemiology of yaws in the Solomon Islands. Briefly, we undertook a survey in Western and Choiseul provinces of the Solomon Islands in September and October 2013. Twenty-five clusters were randomly selected in each province. In each cluster, thirty households were visited, and children aged five to fourteen were enrolled in the study. The study team collected information on yaws symptoms, signs and treatment history. Venepuncture was performed and a serum sample was collected from all participants. Sera were kept on wet ice (4°C) in the field and transferred within 5 days of collection to the National Referral Hospital, Honiara, where they were frozen. Samples were shipped on dry-ice to the London School of Hygiene & Tropical Medicine (LSHTM).

### Sample Size

We assumed that the prevalence of yaws sero-positivity by the gold standard assay would be 30%. We therefore calculated that a sample size of 415 was required to be 80% confident that the true sensitivity of the DPP, compared to the gold standard, was 85% or greater [13]. For the purposes of this study, simple random sampling was used to select samples to undergo parallel testing with both the DPP kit and traditional serology.

### Laboratory Testing

Sera were tested using both the TPPA (Mast Diagnostics, Merseyside UK) and a quantitative RPR (Deben Diagnostics, Sheffield UK) at LSHTM by an operator masked to clinical findings. A second operator, masked to clinical findings and gold standard serology, tested samples using the DPP test kit. Samples for which the control line did not appear were repeated. The manufacturer's instructions were followed for all test kits.

### Statistical Analysis

The sensitivity, specificity, positive and negative predictive values of the DPP test kit were calculated using traditional serology as the gold standard. The DPP-T1 line was assessed against TPPA and the DPP-T2 line was assessed against RPR. Secondary analyses estimated these performance characteristics by RPR titre and presence or absence of clinical signs of yaws. Confidence intervals were calculated using robust standard errors to allow for clustering. Findings are reported in line with the STARD checklist for studies of diagnostic accuracy [14].

### Ethics

Written, informed consent was obtained from the head of each household, who was the parent or guardian of children enrolled in the study, and assent was obtained from all children. Ethical approval for the study was granted by the ethics committees of the Ministry of Health and Medical Services in the Solomon Islands, and LSHTM in the UK.

## Results

### Participant Characteristics

**Figure 1:**
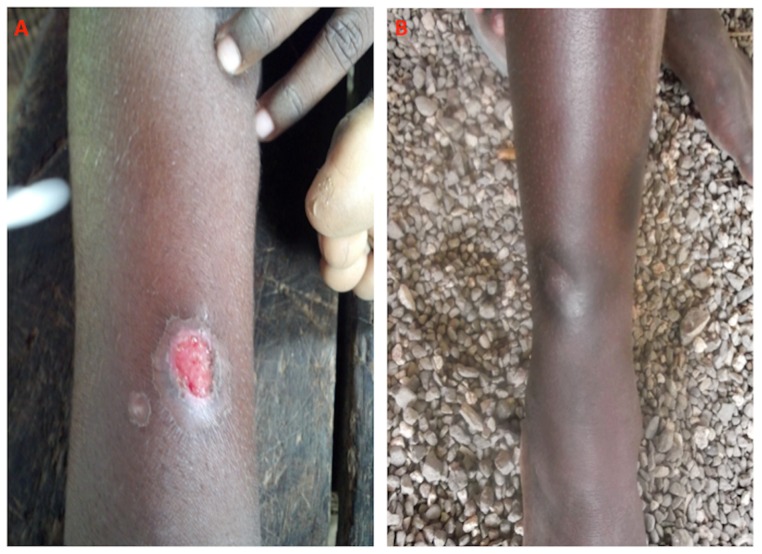
Clinical lesions of yaws. a) Primary yaws ulcer. b) Healed yaws lesion. Images credit: Michael Marks.

Four hundred and fifteen samples were randomly selected. The median age was 9, and 52.1% of participants were male. Individuals selected for this study did not differ significantly from the larger prevalence survey population with regards to demographic or clinical features (data not shown). Clinical findings consistent with active and healed yaws were found in 19 (4.7%) and 34 (8.2%) respectively of the 415 participants (Table 1 and Figure 1).

**Table 1:** Participant characteristics.

Number of children	415
Gender: Male(%)	216 (52.1%)
Age (yrs): Median (IQR)	9 (7–11)
Clinical signs of primary yaws	12 (2.9%)
Clinical signs of secondary yaws	7 (1.7%)
Clinical signs of healed yaws	34 (8.2%)
Recent treatment for yaws	54 (13.0%)

### Laboratory Testing

**Figure 2:**
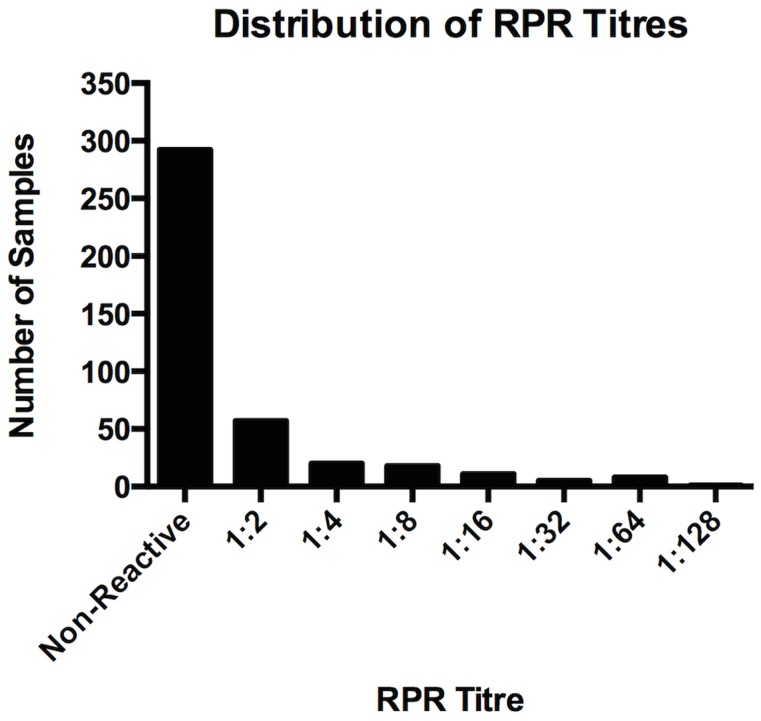
RPR titre distribution.

123 (29.6%) individuals had a reactive TPPA. 120 (28.9%) individuals had a reactive RPR at any titre. By gold standard serology there were 18 individuals with a false positive RPR (defined as a positive RPR and negative TPPA). All false positive RPRs in our study had an RPR titre of 1∶2. The overall prevalence of true RPR reactivity was therefore 102/415 (24.6%). The distribution of RPR titres in the study population is given in Figure 2.

79 individuals (19.0%) had a positive T1(treponemal) line on the DPP kit. 64 (15.4%) individuals had a positive T2 (non-treponemal) line on the DPP kit. There were ten individuals with a positive T2 line but a negative T1 line. The overall prevalence of dual-positivity was therefore 54/415 (13.0%).

The sensitivity of the T1 line against TPPA was 58.5% and the specificity was 97.6%. The positive predictive value was 91.1% and the negative predictive 84.5%. The sensitivity of the T2 line against RPR was 41.7% and the specificity was 95.2%. The positive predictive value was 78.1% and the negative predictive value was 79.9%. The sensitivity of combined T1 and T2 against combined TPPA and RPR was 47.1% and the specificity was 98.1%. The sensitivity of the DPP was strongly related to the RPR titre (Tables 2 and 3)

**Table 2:** Test characteristics.

	Positive by Gold Standard	DPP Sensitivity	DPP Positive Predictive Value	DPP Specificity	DPP Negative Predictive Value
T1 (Treponemal Line) vs TPPA	123	58.5% (43.4–72.2%)	91.1%	97.6%(95.2–98.8%)	84.5%
T2 (Non-Treponemal Line) vs RPR	120	41.7% (28.0–56.8%)	95.2%	95.2%(92–95%)	79.9%
Dual Positive DPP (T1+T2) vs nDual Positive Gold-Standard Serology (TPPA+RPR)	102	47.1%(31.1–63.6%)	88.9%	98.1%(95.2–99.2%)	85.0%

**Table 3:** Test characteristics by RPR titre.

		Positive by Gold Standard	DPP Sensitivity	DPP Specificity
T2 (Non-Treponemal Line) vs RPR	RPR≥1/4	63	61.9%(44.8–76.5%)	92.8%(87.3–96.1%)
	RPR≥1/8	43	72.1%(51.4–86.3%)	91.1%(84.6–95.0%)
	RPR≥1/16	25	92.0% (66.6–98.5%)	89.4%(82.6–93.8%)
Dual Positive DPP (T1+T2) vs Dual Positive Gold-Standard Serology (TPPA+RPR)	RPR≥1/4	63	58.7%(41.3–74.2%)	95.2%(89.7–97.8%)
	RPR≥1/8	43	69.8%(48.3–85.1%)	93.5%(87.2–96.9%)
	RPR≥1/16	25	92.0%(66.6–98.5%)	92.1%(85.2–95.9%)

In individuals with clinical signs of active yaws the sensitivity of the T1 line, compared to TPPA, was 81.8% and the specificity was 100%. The sensitivity of the T2, compared to RPR, was 55.6% and the specificity was 83.3%. A similar association was seen between T2 sensitivity and RPR titre as in the overall population (data not shown).

## Discussion

In this study, we found that in a community surveillance setting, the sensitivity and specificity of the DPP rapid diagnostic test were markedly lower than in the only previous evaluation of the assay for use in clinically active yaws [15]. There was a strong association between the sensitivity of both the T2 line (against gold standard RPR) and RPR titre and also between the T1 line (against gold standard TPPA) and RPR titre. This finding might suggest that the apparent reduced sensitivity of the test reflects, at least in part, lower antibody titres in this population (where positive serology predominantly reflected asymptomatic latent cases) compared to populations in which the assay has previously been evaluated (where there were greater numbers of patients with active clinical disease), rather than a difference in test characteristics per se. Despite the reduced sensitivity and specificity the positive predictive values of the test remained relatively high, reflecting the high prevalence of treponemal infection in this endemic setting.

An association between the sensitivity of the T2 line and the RPR titre has previously been noted [12], but an association between RPR titre and T1 sensitivity has not been described before. It is possible that, in keeping with its lower pathogenicity compared to syphilis, yaws elicits less vigorous antibody production than does its venereal cousin, affecting both the non-specific (non-treponemal) and specific (treponemal) components of that response. ‘Attenuated yaws’ has previously been described in the Solomon Islands [16] with less florid clinical manifestations than noted elsewhere. Widespread use of antibiotics with treponemocidal activity has been postulated as one possible explanation for this postulated clinical entity. It is conceivable that this phenomenon could also contribute to the predominantly low-titre range of antibody responses seen in this study.

The setting in which this evaluation was carried out varies markedly from previous evaluations of the DPP test. In the largest published study, which evaluated the test in the diagnosis of syphilis, Yin and colleagues [11] evaluated the test in a population of 1,323 individuals presenting to a sexual health clinic in China, and found it to have a sensitivity of approximately 95% against TPPA and 86% against RPR. An association between RPR titre and sensitivity was also found in this study, although the performance of the test was better at titres of 1∶4 and 1∶8 than reported here.

Our study has a number of limitations. First, relatively few individuals tested had clinical evidence of active yaws, reflecting the community surveillance setting in which the test was evaluated. Whilst this limits our ability to comment on the value of the test for the purpose of case-confirmation, the aim of this study was to evaluate the DPP's use in screening whole communities at risk of yaws, and for this context we provide the first published data. Second, testing was performed in a central laboratory facility not in the field. Although the test can be performed rapidly (approximately 15–20 minutes per RDT), field-testing was not practical alongside the other activities being performed in our study, for which teams had to move house-to-house. Evaluations of the test elsewhere show that test performance is unaffected by whether venepuncture or finger-prick samples are used [11]. Further evaluations of the test in the field are warranted.

This study has implications for the use of the DPP RDT in yaws surveillance and control. Whilst the sensitivity of the test was lower than previously reported, the specificity remained high, and the negative and positive predictive values of the test were also high. Our data suggest that the DPP test can be used as part of a community surveillance strategy to identify individuals who are dually sero-positive with high-titre RPRs. These individuals are most likely to represent the major source of ongoing transmission. Identification of communities in which such individuals live is vital to allow adequate community targeted treatment to be undertaken [4]. The performance of the test in individuals with clinical evidence of yaws was better than in those without clinical evidence of disease, an association that is likely to be explained by the higher RPR titres found in individuals with clinically active disease. Because of inadequate access to point-of-care diagnostics, many national yaws surveillance systems only report clinically suspected cases. Wider access to DPP testing could allow a larger proportion of these cases to be evaluated serologically, which would both critically refine understanding of global yaws epidemiology as it evolves towards the eradication endpoint, but also provide a vital clinical aid in the post-MDA setting, where many conditions mimicking yaws will continue to present to health-care facilities. Further evaluation of the DPP in other yaws surveillance settings would be welcomed to provide the basis for guideline development.

## Supporting Information

Checklist S1 STARD checklist.
(DOC)

Dataset S1 Serology and DPP results.
(XLS)

Figure S1 STARD flowchart.
(TIFF)
