# Supplementary figures and images for: Evaluation of a Rapid Diagnostic Test for Yaws Infection in a Community Surveillance Setting
Source: PLoS Negl Trop Dis. 2014 Sep 11;8(9):e3156. doi: 10.1371/journal.pntd.0003156 (PMC4161315; doi:10.1371/journal.pntd.0003156)

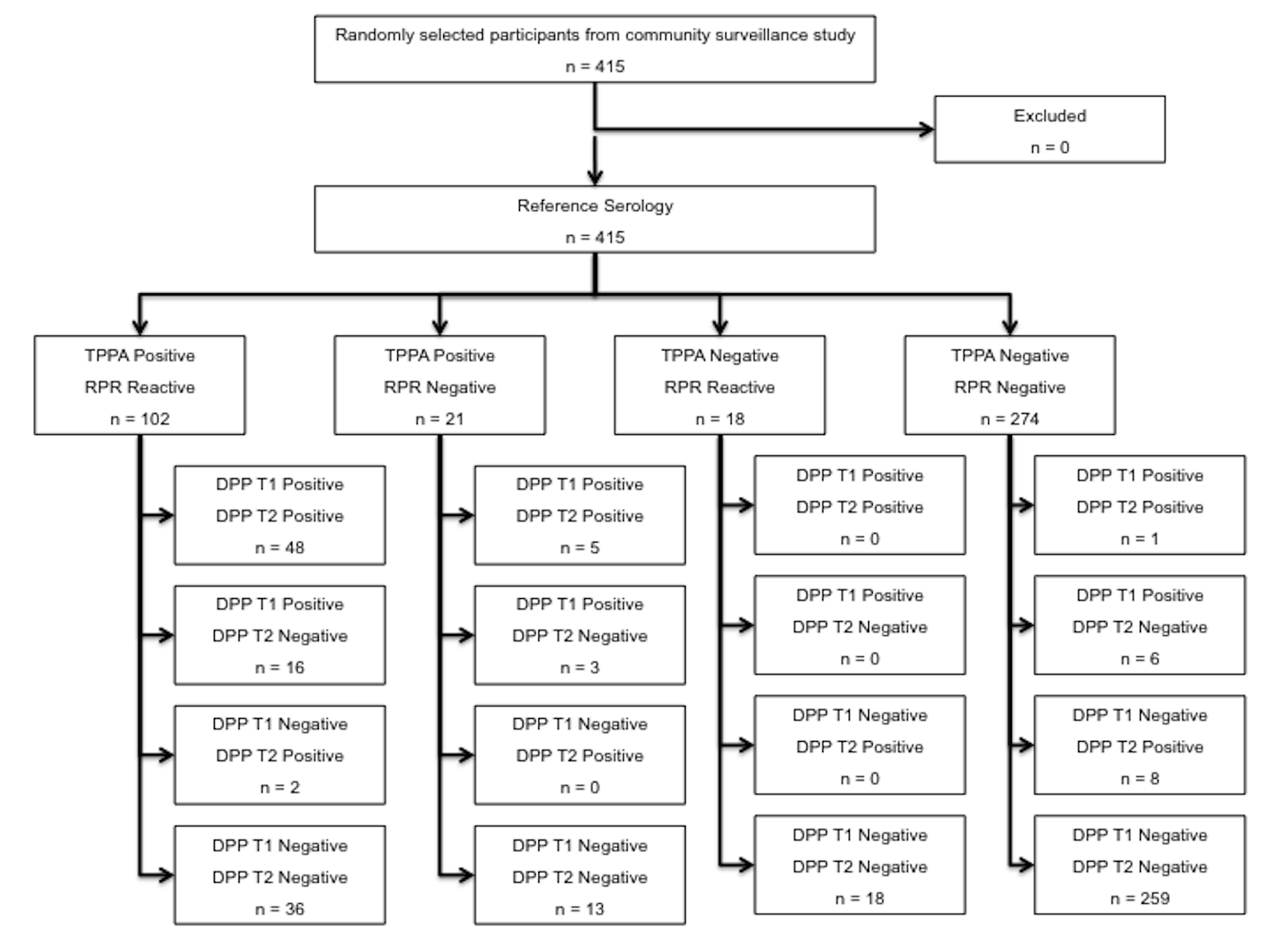

Supplement: Figure S1 — STARD flowchart. (TIFF) [file pntd.0003156.s003.tiff]
